# 外周血淋巴细胞亚群在初治多发性骨髓瘤患者中的预后价值

**DOI:** 10.3760/cma.j.cn121090-20240830-00329

**Published:** 2025-06

**Authors:** 召云 刘, 祥鸿 赵, 惠 刘, 凯 丁, 凤平 彭, 凤娟 姜, 蓉 付

**Affiliations:** 天津医科大学总医院血液内科，天津市骨髓衰竭及癌性造血克隆防治重点实验室，天津市血液病研究所，天津 300052 Department of Hematology, Tianjin Medical University General Hospital, Tianjin Key Laboratory of Bone Marrow Failure and Malignant Hemopoietic Clone Control, Tianjin Institute of Hematology, Tianjin 300052, China

**Keywords:** 多发性骨髓瘤, 淋巴细胞亚群, 预后, 免疫风险评分, Multiple myeloma, Lymphocyte subsets, Prognosis, Immune risk score

## Abstract

**目的:**

探索外周血淋巴细胞亚群在初治多发性骨髓瘤（NDMM）患者中的预后价值。

**方法:**

回顾性分析2017—2022年天津医科大学总医院收治的NDMM患者133例，通过LASSO回归从外周血淋巴细胞亚群中筛选预测亚群，通过受试者工作特征曲线分析计算最佳截断值，基于多因素分析结果构建列线图，通过一致性指数和校准曲线评估列线图的预测性能。采用Kaplan-Meier曲线和秩和检验比较免疫风险评分高危与低危组患者总生存（OS）期和无进展生存（PFS）期的差异。

**结果:**

通过LASSO回归筛选出CD16^+^CD56^+^ NK细胞的百分比和绝对计数、CD3^+^ T淋巴细胞、CD3^+^CD8^+^ T淋巴细胞、CD3^-^ CD19^+^ B淋巴细胞的绝对计数作为预测亚群，根据各淋巴细胞亚群系数计算NDMM患者的免疫风险评分，免疫风险评分的曲线下面积为0.737，最佳截断值为−1.834，据此将患者分为高危组和低危组。生存分析显示，免疫风险评分高危与低危组患者的3年OS率差异有统计学意义（87.4％对49.0％，*P*<0.001），且在微小残留病阴性患者中，免疫风险评分高危与低危组患者3年OS率的差异有统计学意义（100％对68.6％，*P*＝0.001）。多因素分析显示，血清钙（*P*＝0.034）、高危细胞遗传学异常（*P*＝0.002）和免疫风险评分（*P*<0.001）为NDMM患者的预后影响因素，以此构建列线图。列线图的一致性指数为0.793，校准曲线显示其预测能力良好，列线图可以对不同预后分期系统的危险度进行精准划分。

**结论:**

外周血淋巴细胞亚群联合分析对于判断NDMM患者的预后具有重要价值。

## Abstract (en)

**Objective:**

To explore the prognostic value of peripheral blood lymphocyte subsets in patients with newly diagnosed multiple myeloma（NDMM）.

**Methods:**

The study retrospectively analyzed 133 patients with NDMM admitted to the General Hospital of Tianjin Medical University General Hospital between 2017 and 2022. The least absolute shrinkage and selection operator（LASSO）regression was used to screen the predictive subgroups from the peripheral blood lymphocyte subsets, and the optimal cutoff value was calculated through receiver operating characteristic curve analysis. A nomogram was constructed based on the results of the multiple-factor analysis, and the predictive performance of the nomogram was evaluated by the concordance index and calibration curve. Kaplan-Meier curves and log-rank tests were conducted to compare the differences in overall survival（OS）and progression-free survival between the high-risk and low-risk immune risk scores groups.

**Results:**

Using LASSO regression, the percentages and absolute counts of CD16^+^CD56^+^ NK cells, CD3^+^ T lymphocytes, CD3^+^CD8^+^ T lymphocytes, and CD3^-^CD19^+^ B lymphocytes were selected as predictive subgroups. The immune risk score of patients with NDMM was calculated based on the coefficients of each lymphocyte subgroup. The area under the curve of the immune risk score was 0.737, and the optimal cutoff value was −1.834. Based on this, the patients were divided into high-risk and low-risk groups. Survival analysis showed a significant difference in the 3-year OS rate between the high-risk and low-risk immune risk score groups（87.4％ *vs* 49.0％, *P*<0.001）, and a significant difference in the 3-year OS rate between the high-risk and low-risk immune risk score groups in patients with minimal residual disease negative（100％ *vs* 68.6％, *P*＝0.001）. Multivariate analysis showed that serum calcium（*P*＝0.034）, high-risk cytogenetic abnormalities（*P*＝0.002）, and immune risk score（*P*<0.001）were prognostic factors for patients with NDMM, and a nomogram was constructed based on these factors. The consistency index of the nomogram was 0.793, and the calibration curve showed good predictive ability. The nomogram can accurately classify the risk of different prognostic staging systems.

**Conclusion:**

The combined analysis of lymphocyte subsets in the peripheral blood has an important value in predicting the prognosis of patients with NDMM.

多发性骨髓瘤（MM）是一种以恶性克隆性浆细胞异常增殖为特点的恶性血液系统疾病。其发病机制复杂，除肿瘤细胞自身克隆性增殖外，骨髓抑制性免疫微环境的形成也是导致疾病发生发展的重要原因[1]–[4]。寻找有效的生物标志物，精准评价患者的预后有助于制定科学的治疗策略以提升疗效[5]。目前常用的MM患者预后分期系统包括国际分期系统（ISS）、修订的国际分期系统（R‑ISS）、国际分期系统第二次修订版（R2‑ISS），这些分期系统主要聚焦于肿瘤负荷与细胞遗传学异常对MM患者预后的影响，忽略了免疫微环境对预后的影响。免疫紊乱是MM患者重要的发病机制之一，已有报道显示，MM患者存在免疫细胞数量和功能缺陷且与患者的预后相关[4],[6]。有研究显示，CD3^+^CD4^+^和CD3^+^CD8^+^ T细胞与MM患者的预后相关[7]。此外，进展期MM患者NK细胞数量下降[8]–[9]。有研究显示，MM患者的免疫微环境与其R-ISS分期相关，免疫指标结合ISS分期可以提高对MM患者预后的预测能力[10]–[11]。本研究拟通过流式细胞术检测初治MM（NDMM）患者外周血淋巴细胞亚群，筛选出对MM患者预后有影响的生物标志物。

## 病例与方法

1. 病例：本研究纳入2017—2022年天津医科大学总医院收治的NDMM患者133例，MM的诊断标准符合国际骨髓瘤工作组（IMWG）2014年诊断标准[12]，并分别计算每例患者的ISS[13]、R-ISS[14]和R2-ISS[15]分期。每例患者至少接受过1种蛋白酶体抑制剂（PI）、免疫调节剂（IMID）或CD38单抗治疗。本研究中执行的所有程序均符合《赫尔辛基宣言》（2013年修订版）并经天津医科大学总医院伦理委员会批准（IRB2022-WZ-187）。

2. 临床资料：收集患者的临床信息，包括年龄、性别、HGB、血清钙、血清肌酐、血清β_2_-微球蛋白、血清乳酸脱氢酶（LDH）和细胞遗传学信息。其中高危细胞遗传学异常指间期荧光原位杂交检测出del（17p）、t（4;14）、t（14;16）[14]。

3. 外周血淋巴细胞亚群检测：应用BD FACS™裂解液（美国BD公司产品）裂解外周血样本，应用美国BD公司的TBNK六色免疫荧光试剂（CD3 FITC/CD16 PE+CD56 PE/CD45 PerCP-Cy™5.5/CD4 PE-Cy™7/CD19 APC/CD8 APC-Cy™7）通过流式细胞术测定外周血淋巴细胞亚群。使用BD绝对计数管（美国BD公司产品）计算外周血淋巴细胞亚群中CD3^+^T细胞、CD3^+^CD8^+^T细胞、CD3^+^CD4^+^T细胞、CD16^+^CD56^+^NK细胞及CD3^-^ CD19^+^B细胞的绝对计数并计算其占总细胞数的百分比。

4. 微小残留病（MRD）检测：采用基于欧洲流式联盟（EuroFlow）多参数流式细胞术方案分析MM患者骨髓样本的MRD（CD38-FITC、CD56-PE、CD45-PerCPCy5.5、CD19-PECy7、CD27-BV510、CD138-BV421、CyIgκ-APC、CyIgλ-APC-Cy7）[16]。共90例MM患者进行了MRD检测，检测敏感度为10^−5^。

5. 随访：随访截止日期为2023年6月1日。通过查阅住院电子病历及电话进行随访，中位随访时间为25（1～71）个月。

6. 统计学分析：分类变量用例数（％）描述，不符合正态分布的连续变量以*M*（范围）描述。使用SPSS 26.0和R 4.0.0进行统计学分析。组间比较采用*χ*^2^检验。采用LASSO-Cox回归筛选危险因素。风险评分的计算公式为各变量LASSO回归系数×变量值之和。利用受试者工作特征（ROC）曲线及其曲线下面积（AUC）计算最佳截断值。采用单因素及多因素Cox比例风险回归模型寻找影响预后的独立危险因素。利用Kaplan-Meier法绘制生存曲线，利用秩和检验比较生存差异。C指数用于比较不同模型的预测性能。*P*<0.05为差异有统计学意义。

## 结果

1. 临床特征：研究共纳入133例患者。133例患者中，男74例（55.6％），女59例（44.4％），年龄≥65岁患者63例（47.4％）。88例（66.2％）患者初诊时HGB<100 g/L，26例（19.5％）患者血钙>2.75 mmol/L。41例（30.8％）患者出现肾功能损害。71例（53.3％）患者β_2_-微球蛋白≥5.5 mg/L，32例（24％）患者LDH升高。此外，32例（24％）患者出现高危细胞遗传学异常。

2. 外周血淋巴细胞亚群对患者预后的预测价值：本研究纳入NDMM患者外周血CD3^+^ T淋巴细胞、CD3^+^CD4^+^ T淋巴细胞、CD3^+^CD8^+^ T淋巴细胞、CD3^-^ CD19^+^ B淋巴细胞和CD16^+^CD56^+^ NK细胞的绝对计数和百分比，利用LASSO-Cox回归模型筛选影响NDMM患者预后的因素。共筛选出5个因素：CD16^+^CD56^+^ NK细胞绝对计数和百分比，CD3^+^ T淋巴细胞、CD3^+^CD8^+^ T淋巴细胞和CD3^-^ CD19^+^B淋巴细胞的绝对计数，并根据其系数得出免疫风险评分的计算公式：免疫风险评分＝−0.032 583 3×CD16^+^CD56^+^ NK细胞百分比−0.001 033 182×CD3^+^ T淋巴细胞绝对计数−0.001 568 054×CD3^+^CD8^+^ T淋巴细胞绝对计数−0.000 070 896 14×CD16^+^CD56^+^ NK细胞绝对计数+0.001 678 835×CD3^-^ CD19^+^ B淋巴细胞绝对计数。ROC曲线分析显示，免疫风险评分的AUC为0.737（*P*<0.001，最佳截断值：−1.834）（图1）。根据最佳截断值将患者分为高危组（评分≥−1.834，38例）与低危组（评分<−1.834，95例）。高危组与低危组的患者临床特征见表1，低危组患者的预后优于高危组［3年总生存（OS）率：87.4％对49.0％，*P*<0.001；3年无进展生存（PFS）率：70.1％对34.3％，*P*<0.001］（图2A、B）。将MRD与免疫风险评分结合起来进一步分析，并根据MRD状态与免疫风险评分将90例患者分为4组，低免疫风险评分伴MRD阴性、高免疫风险评分伴MRD阴性、低免疫风险评分伴MRD阳性、高免疫风险评分伴MRD阳性MM患者的3年OS率分别为100％、68.6％、90.5％和63.5％（*P*＝0.001）（图3A），3年PFS率分别为94.1％、57.1％、57.9％、40.3％（*P*＝0.004）（图3B）。

**图1:**
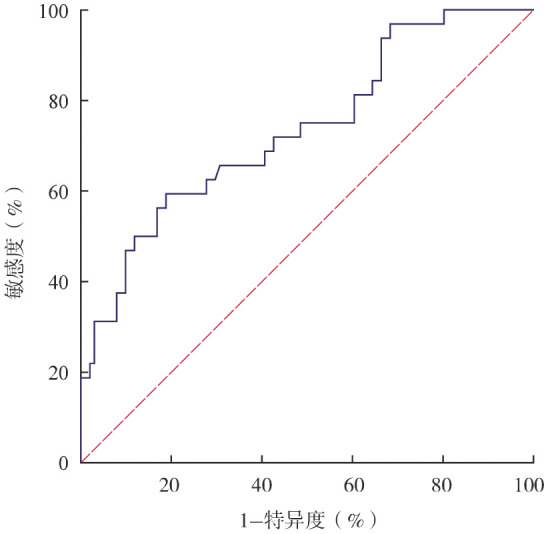
免疫风险评分预测初治多发性骨髓瘤患者危险度的受试者工作特征曲线

**表1:** 免疫风险评分低危组与高危组多发性骨髓瘤患者的临床特征［例（％）］

特征	低危组（95例）	高危组（38例）	*χ*^2^值	*P*值
年龄			0.591	0.442
<65岁	52（54.7）	18（47.4）		
≥65岁	43（45.3）	20（52.6）		
性别			0.003	0.956
男	53（55.8）	21（55.3）		
女	42（44.2）	17（44.7）		
HGB			1.343	0.246
≥100 g/L	35（36.8）	10（26.3）		
<100 g/L	60（63.2）	28（73.7）		
血清钙浓度			13.430	<0.001
≤2.75 mmol/L	84（88.4）	23（60.5）		
>2.75 mmol/L	11（11.6）	15（39.5）		
血肌酐			14.900	<0.001
<177 µmol/L	75（78.9）	17（44.7）		
≥177 µmol/L	20（21.1）	21（55.3）		
血清β_2_-微球蛋白			4.834	0.028
<5.5 mg/L	50（52.6）	12（31.6）		
≥5.5 mg/L	45（47.4）	26（68.4）		
血白蛋白			0.113	0.737
≥35 g/L	37（38.9）	16（42.1）		
<35 g/L	58（61.1）	22（57.9）		
血LDH			1.646	0.199
<250 U/L	75（78.9）	26（68.4）		
≥250 U/L	20（21.1）	12（31.6）		
高危细胞遗传学异常			0.004	0.949
阴性	72（75.8）	29（76.3）		
阳性	23（24.2）	9（23.7）		
治疗方式			2.053	0.152
PI/IMID	86（90.5）	31（81.6）		
CD38抗体	9（9.5）	7（18.4）		
auto-HSCT			0.608	0.435
否	83（87.4）	35（92.1）		
是	12（12.6）	3（7.9）		

**注** PI：蛋白酶体抑制剂；IMID：免疫调节剂；auto-HSCT：自体造血干细胞移植

**图2:**
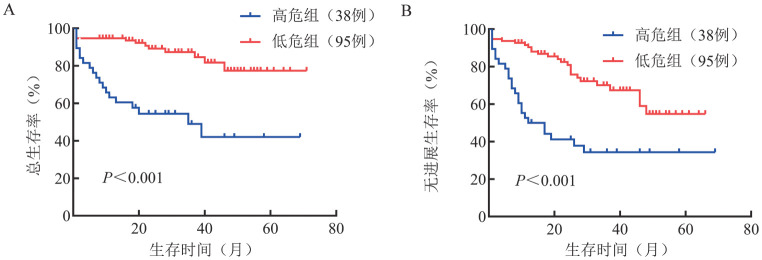
免疫风险评分高危组和低危组初治多发性骨髓瘤患者的总生存（A）及无进展生存（B）曲线

**图3:**
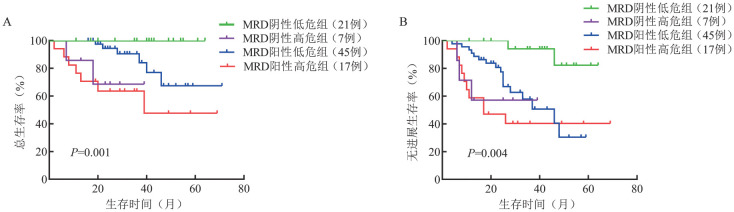
不同免疫风险评分及微小残留病（MRD）情况初治多发性骨髓瘤患者的总生存（A）及无进展生存（B）曲线

3. 免疫风险评分联合临床指标对患者预后的预测价值：多因素回归分析显示，血清钙浓度（*P*＝0.034）、高危细胞遗传学异常（*P*＝0.002）和免疫风险评分（*P*<0.001）是影响NDMM患者预后的独立危险因素（表2），据此构建列线图。列线图的C指数为0.793，其预测能力优于单一指标（免疫风险评分、血清钙浓度和高危细胞遗传学异常的C指数分别为0.737、0.636和0.611）。校准曲线显示，1年、2年及3年OS率预测值与实际值一致性较好。时间依赖的ROC曲线AUC值分别为：1年OS率：0.835（95％ *CI*：0.734～0.935），2年OS率：0.836（95％ *CI*：0.738～0.935），3年OS率：0.846（95％ *CI*：0.752～0.940）。根据列线图得分的中位数将患者分为高危组和低危组，进一步比较各预后分期系统不同分期低危组和高危组患者的OS率和PFS率：ISS Ⅰ、Ⅱ期的3年OS率：97.2％对63.6％，*P*＝0.003；ISS Ⅰ、Ⅱ期的3年PFS率：71.6％对51.9％，*P*＝0.049。ISS Ⅲ期3年OS率：88.4％对56.9％，*P*＝0.030；ISS Ⅲ期3年PFS率：74.1％对43.8％，*P*＝0.029。R-ISS Ⅰ、Ⅱ期的3年OS率：89.6％对63.9％，*P*＝0.001；R-ISS Ⅰ、Ⅱ期3年PFS率：72.1％对58.2％，*P*＝0.019。R-ISS Ⅲ期3年OS率：66.7％对47.9％，*P*＝0.348；3年PFS率：71.1％对30.6％，*P*＝0.351。R2-ISS Ⅰ、Ⅱ期3年OS率：94.7％对57.1％，*P*＝0.044；R2-ISS Ⅰ、Ⅱ期3年PFS率：88.0％对43.2％，*P*＝0.012。R2-ISS Ⅲ、Ⅳ期3年OS率：92.6％对60.5％，*P*＝0.003；R2-ISS Ⅲ、Ⅳ期3年PFS率：64.2％对48.1％，*P*＝0.033。

**表2:** 影响多发性骨髓瘤患者总生存的预后因素分析

预后因素	单因素分析		多因素分析	
	*HR*（95％ *CI*）	*P*值	*HR*（95％ *CI*）	*P*值
年龄（≥65岁，<65岁）	1.937（0.951～3.947）	0.069		
性别（女性，男性）	1.009（0.504～2.023）	0.979		
HGB（<100 g/L，≥100 g/L）	1.427（0.590～3.452）	0.430		
血清钙浓度（>2.75 mmol/L，≤2.75 mmol/L）	3.399（1.674～6.900）	0.001	2.233（1.064～4.686）	0.034
血肌酐（≥177 µmol/L，<177 µmol/L）	1.895（0.942～3.812）	0.073		
血清β_2_-微球蛋白（≥5.5 mg/L，<5.5 mg/L）	2.194（1.038～4.639）	0.040	1.589（0.732～3.447）	0.241
血白蛋白（<35 g/L，≥35 g/L）	1.176（0.575～2.408）	0.657		
血LDH（≥250 U/L，<250 U/L）	1.455（0.689～3.073）	0.326		
高危细胞遗传学异常（有，无）	2.764（1.352～5.652）	0.005	3.300（1.570～6.937）	0.002
免疫风险评分（≥−1.834，<−1.834）	2.961（1.807～4.853）	<0.001	2.793（1.635～4.773）	<0.001

## 讨论

MM目前仍是一种无法治愈的疾病，免疫失调在MM的发病和进展中发挥重要作用[17]–[18]，MM患者抑制性免疫微环境可以促进肿瘤的发生发展[8],[18]–[22]。改善抑制性免疫微环境、恢复抗肿瘤免疫反应已成为当代MM治疗的重要方向，因此，需要发掘新的免疫学标志物预测MM患者的预后[5]。

MM患者的免疫状态与患者的预后相关[4]。我们回顾性分析了133例NDMM患者淋巴细胞亚群的百分比、绝对计数及临床特征，发现CD3^+^ T淋巴细胞绝对计数高、CD16^+^CD56^+^ NK细胞绝对计数及百分比高，CD8^+^ T淋巴细胞绝对计数高及CD19^+^ B淋巴细胞绝对计数低的患者预后更好。CD8^+^ T淋巴细胞参与免疫监视并杀伤肿瘤细胞[23]–[24]。有研究报道，肿瘤特异性CD8^+^ T淋巴细胞与MM患者的预后相关[9]。NK细胞参与固有免疫，可以释放穿孔素和颗粒酶杀伤MM细胞[25]–[28]，此外，还可以刺激树突状细胞和T细胞的免疫反应[25]–[26],[29]。研究还观察到，进展期MM患者外周血NK细胞绝对计数下降[4],[9]及CD19^+^ B淋巴细胞绝对计数升高的MM患者预后更佳，后者与本研究结果相反[30]。但也有报道显示，MM患者外周血克隆性CD19^+^细胞绝对计数低与患者耐药性相关[31]–[32]。CD4^+^T细胞被认为与MM患者的预后有关[33]，但本研究并未发现其对预后的影响，上述结果还有待进一步证实。

MRD是评价MM患者疗效的重要指标[34]–[35]。MM患者的免疫微环境与其MRD状态有关[36]–[39]，将免疫分析与MRD结合有助于识别深度缓解的患者。本研究显示，免疫风险评分低且MRD阴性的患者获得了最深程度的缓解。MRD联合外周血淋巴细胞亚群可能有助于更全面地判断MM患者的疗效。

目前的MM风险分层系统主要集中于特定的分子和细胞遗传学异常，忽略了免疫微环境对预后的影响[11],[40]。免疫紊乱与MM的发病及进展有关。一项研究发现，在高危细胞遗传学异常和（或）ISS Ⅲ期MM患者中，CD4/CD8比值升高且总T细胞百分比低[40]。此外，CD8^+^ T细胞百分比与MM患者的R-ISS分期有关[11]。本研究显示，免疫风险评分是NDMM患者的预后影响因素，免疫风险评分结合临床指标构建的列线图显示出更好的预测能力。列线图可对ISS、R2-ISS和R-ISS分期体系中不同分期MM患者的危险度进一步分层。本研究显示，外周血淋巴细胞亚群联合分析对MM患者的预后有判断价值，外周血淋巴细胞亚群联合患者临床特征及细胞遗传学异常可进一步区分不同危险度的患者，尤其是分期体系的中低危患者。在R-ISS Ⅲ期患者中未显示差异有统计学意义，待增加样本量进一步分析。

本研究有一定的局限性。首先，单中心研究样本量小，治疗上虽在亚组分析时对治疗因素进行了匹配，但缺少严格意义上治疗方案的匹配设计，有待将来进行大样本量的多中心、前瞻性研究；其次，本研究忽略了其他可能影响预后的细胞亚群，如间充质干细胞和树突状细胞等免疫细胞的功能变化情况，亦需要将来深入探讨。

综上所述，外周血淋巴细胞亚群是NDMM患者的预后影响因素。此外，结合临床特征进行分析可以进一步细化不同危险度MM患者的预后分层。
